# Music-instruction intervention for treatment of post-traumatic stress disorder: a randomized pilot study

**DOI:** 10.1186/s40359-018-0274-8

**Published:** 2018-12-19

**Authors:** L. E. Pezzin, E. R. Larson, W. Lorber, E. L. McGinley, Timothy R. Dillingham

**Affiliations:** 1 Department of Medicine and Center for Patient Care and Outcomes Research, Medical College of Wisconsin, Milwaukee, WI USA; 2 Zablocki Veterans Administration Medical Center, Milwaukee, WI USA; 3 Department of Psychiatry and Behavioral Medicine, Medical College of Wisconsin, Milwaukee, USA; 4 The William J. Erdman II, Professor and Chair, Department of Physical Medicine and Rehabilitation, University of Pennsylvania, 1800 Lombard St. First Floor, Philadelphia, PA 19146 USA

**Keywords:** Post-traumatic stress disorder, Depression, Randomized trial

## Abstract

**Background:**

Post-traumatic Stress Disorder (PTSD) is a common sequelae of severe combat-related emotional trauma that is often associated with significantly reduced quality of life in afflicted veterans. To date, no published study has examined the effect of an active, music-instruction intervention as a complementary strategy to improve the psychological well-being of veterans with PTSD. The purpose of this study was to examine the feasibility and potential effectiveness of an active, music-instruction intervention in improving psychological health and social functioning among Veterans suffering from moderate to severe PTSD.

**Methods:**

The study was designed as a prospective, delayed-entry randomized pilot trial. Regression-adjusted difference in means were used to examine the intervention’s effectiveness with respect to PTSD symptomatology (primary outcome) as well as depression, perceptions of cognitive failures, social functioning and isolation, and health-related quality of life (secondary outcomes).

**Results:**

Of the 68 Veterans who were self- or provider-referred to the program, 25 (36.7%) were ineligible due to (i) absence of a PTSD diagnosis (*n* = 3); participation in ongoing intense psychotherapy (*n* = 4) or inpatient substance abuse program (*n* = 2); current resident of the Domiciliary (*n* = 8) and inability to participate due to distance of residence from the VA (*n* = 8). Only 3 (4.4%) Veterans declined participation due to lack of interest. The mean age of enrolled subjects was 51 years old [range: 22 to 76]. The majority was male (90%). One-quarter were African American or Black. While 30% report working full or part time, 45% were retired due to disability. Slightly over one-quarter were veterans of the OEF/OIF wars. Estimates from regression-adjusted treatment effects indicate that the average PTSD severity score was reduced by 9.7 points (*p* = 0.01), or 14.3% from pre- to post-intervention. Similarly, adjusted depressive symptoms were reduced by 20.4% (− 6.3 points, *p* = 0.02). There were no statistically significant regression-adjusted effects on other outcomes, although the direction of change was consistent with improvements.

**Conclusions:**

Our findings suggest that the active, music-instruction program holds promise as a complementary means of ameliorating PTSD and depressive symptoms among this population.

**Trial registration:**

Trial registered at ClinicalTrials.gov with protocol number Medical College of Wisconsin PRO00019269 on 11/29/2018 (Retrospectively registered).

## Background

Post-Traumatic Stress Disorder (PTSD) is a common sequelae of severe emotional trauma that is often associated with combat exposure. PTSD has significant implications for quality of life in afflicted veterans, defined by the U.S. Veteran Administration as persons who have served in the active U.S. military, naval or air force and who have been honorably discharged or released. Almost half of all U.S. male Vietnam veterans with current PTSD have been arrested or jailed at least once, 34.2% more than once, and 11.5% had been convicted of a felony [1]. Day-to-day functioning is also adversely impacted by PTSD as indicated in Breslau et al. [2] who found that individuals with full PTSD, compared to those with partial PTSD, demonstrated greater impairment in terms of work days lost, interference with work or daily activities, decreased time spent with people in personal life, and increased conflicts with others because of their reactions to the traumatic experience [3].

PTSD places a particularly significant burden on interpersonal relationships resulting in loneliness and isolation, which may further intensify psychiatric symptoms. Research from the National Co-morbidity Study, for example, indicate that although those with PTSD have the same likelihood as those without PTSD to be married at any point in time, they are 3 to 6 times more likely to divorce. [4] Similarly, about one-third of Veterans with PTSD engaged in intimate partner violence over the one-year observation period compared to 13.5% among veterans without PTSD [4].

Current treatment options for PTSD include psychotherapy, medication management, or both in combination. Psychotherapy approaches with the strongest demonstrated efficacy include cognitive behavioral therapies such as prolonged exposure therapy, stress inoculation training, cognitive processing therapy, eye movement desensitization and reprocessing, and several combinations of these procedures [5–11]. Among the many medications available, none is uniformly successful and all have side effects, underscoring the need for adjuvant means of symptom control that patients can incorporate into a self-management strategy for long term use. Recognizing such need, the U.S. Veterans Administration and the Department of Defense have released a practice guideline stating that Complementary and Alternative Medicine (CAM) may “facilitate engagement in medical care and may be indicated for some patients who refuse evidence-based treatments.”

A number of studies, including five randomized controlled trials, have examined the efficacy of music as a complementary therapy in the treatment of mental illnesses. A recent review [12] indicated that several studies have found greater reductions in symptoms of depression among patients who received music therapy versus standard care for depression [13–16]. To date, however, no published study has examined the effect of an active music-instruction intervention as a complementary strategy to improve the psychological well-being of veterans with PTSD [1].

Filling in this knowledge gap, the purpose of this pilot study was to examine the feasibility and potential effectiveness of an active, music-instruction intervention at improving psychological health and social functioning among a high-risk population of Veterans suffering from moderate to severe PTSD. We hypothesize that the intervention would decrease Veterans*’* PTSD symptomatology, which was the outcome measure of most interest. We also posited that depression and perception of cognitive difficulties would be lessened, and that social functioning and health-related quality of life would be improved.

### Data and methods

#### Study population

The study population consisted of veterans receiving routine care for PTSD symptoms at the Zablocki VA Medical Center in Milwaukee, WI. Eligible Veterans were those who (i) had at least one visit for mental health treatment in the prior six months with a primary diagnosis of PTSD (ICD9CM 309.81–83) and (ii) exhibited moderate to severe PTSD symptoms at the time of enrollment (Posttraumatic Stress Disorder Checklist > = 50) [17]. Veterans were excluded from the study if they were currently participating in an intense psychotherapy program (residential or outpatient) or if they were already receiving guitar lessons from a Guitars for Vets volunteer.

#### Recruitment

Eligible subjects were informed about the study while attending PTSD-related programming via IRB-approved informational flyers that included contact information for study participation. In addition, veterans receiving non-residential services at the VA Domiciliary facility could self-refer to the program, provided that they were not involved in a residential treatment program for PTSD. Eligibility was determined from evidence of PTSD diagnosis from medical records. Finally, a postcard was mailed inviting study participation to potentially eligible veterans who had been identified through the VA medical record system as having a diagnosis of PTSD or who had visits to mental health providers over the past six months. All Veterans that enrolled in the study gave written consent prior to participation.

#### The intervention

This research project took advantage of an established partnership between the Zablocki VA in Milwaukee WI and *Guitars for Vets*, a 501(c)(3) non-profit organization providing Veterans receiving treatment at Veteran’s Administration facilities with guitar instruction by professional music teachers. The intervention was designed as an active intervention and provided veterans with an acoustic guitar, guitar pick and tuning instruments, a music book, practice CDs, and individual and group sessions of music instruction during a six-week intervention period. Six tailored one-hour individual guitar instruction sessions were scheduled (one session per week for six weeks). In addition to the six Veteran-centered, tailored individual lessons, the intervention provided three group sessions. Veterans were given a guitar that they could keep upon completion of the training program. Sessions were offered in the late afternoon and early evenings at the Zablocki VA Domiciliary, which provided an excellent non-clinical environment with ample room for such activities. The same instructor was assigned to a subject for the duration of the study, and group sessions were supervised by the Education Director of Guitars for Vets.

#### Study design

**Fig. 1:**
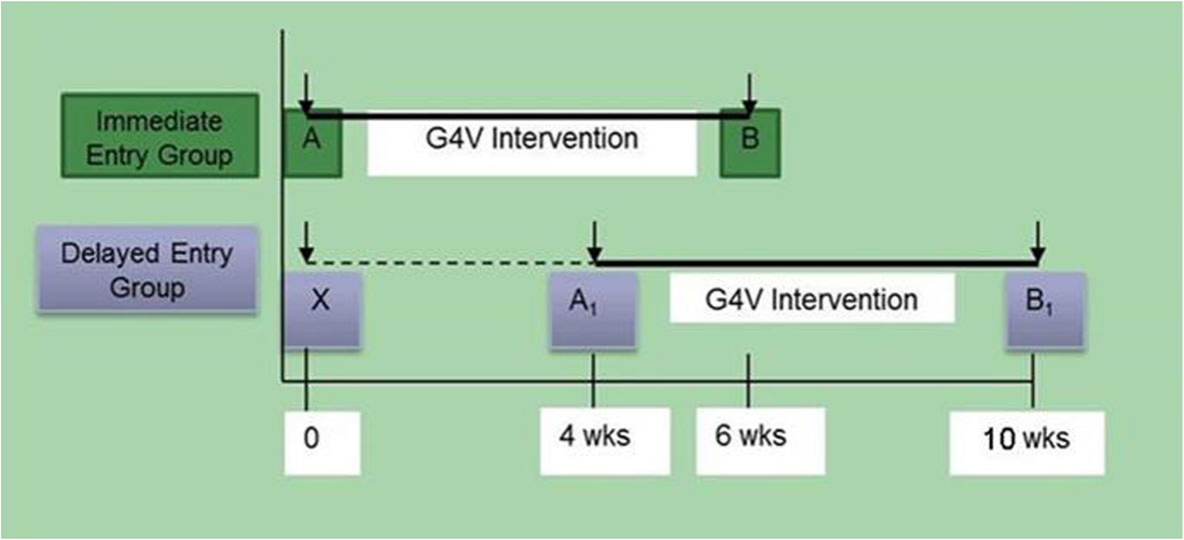
Study Design

**Fig. 2:**
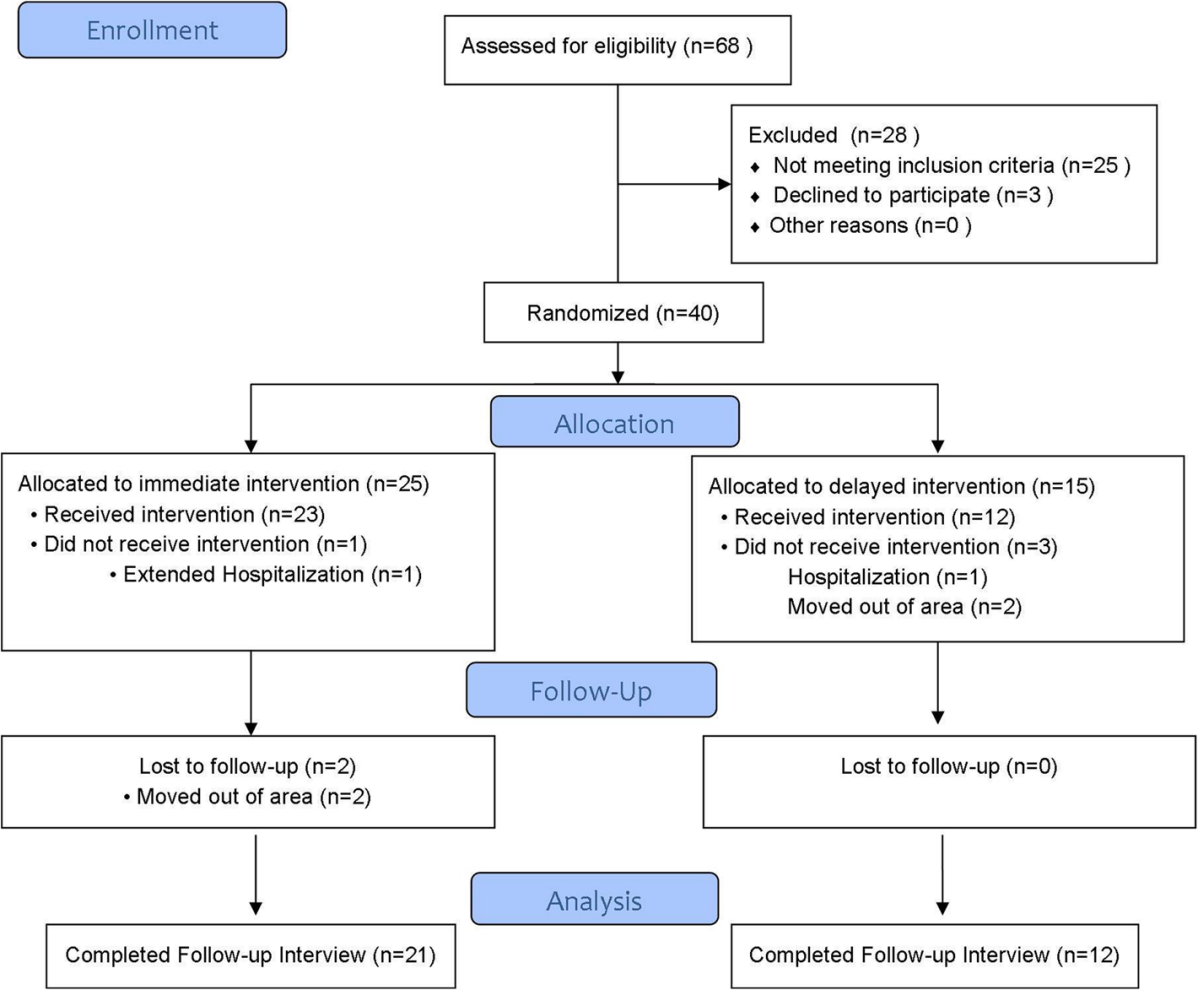
CONSORT Figure

This was a prospective, delayed-entry randomized pilot trial of 40 subjects. Given its pilot nature, a formal power calculation was not performed, although it was estimated that 40 subjects would enable us to detect a 15% or higher reduction between pre-post PCLC scores with 80% power at α = 0.05. The study design is depicted in Fig. 1 with the associated CONSORT flow diagram depicted in Fig. 2. Enrollment occurred after the research associate had completed the initial eligibility assessment and consent. Following eligibility determination and consent process, Veterans were interviewed in-person by a trained interviewer, using a structured survey. Veterans were then randomized to either (1) immediate entry or (2) delayed entry intervention arm using a 2:1 ratio in order to maximize the number of subjects immediately eligible to receive the intervention. In addition, given the expected higher attrition among Veterans randomized to the delayed entry group, the wait period for was set to 4 weeks.

The intervention content and duration was the same across both groups. Following the baseline interview (A), veterans randomized to the *immediate entry* group directly engaged in the intervention described above and were interviewed at the end of the intervention period (B), roughly 6 weeks later. Those randomized to the *delayed entry* group had their baseline interview (X) repeated at the end of the delayed entry period (A_1_) prior to receiving the 6-week intervention as well as after intervention completion (B_1_). This approach enabled us to ascertain the natural history and temporal variation in PTSD symptoms.

#### Variable definitions and measurement

The primary outcome was PTSD symptoms as measured by the PTSD Checklist Civilian (PCLC) [17, 18], a self-report scale that measures PTSD presence and severity. The 17 items correspond to Diagnostic and Statistical Manual DSM-IV symptoms of PTSD. The level of distress produced by each symptom is rated from 1 (not at all) to 5 (extremely). A score > 50 on this measure is considered clinically significant (maximum score = 85). The PCLC has been shown to have good reliability and convergent validity [17].

Secondary outcomes were depression, perceptions of cognitive failures, social functioning, and health-related quality of life. Depression was assessed using the Beck Depression Inventory-II (BDI-II), [19] a 21-item self-report scale measuring the presence and severity of depressive symptoms over the two weeks preceding test administration. Each answer ranges in score from 0 to 3. Total scores indicate minimal (0–13), mild (14–19), moderate (20–28), and severe (29–63; maximum = 63) levels of reported depression. The Cognitive Failures Questionnaire (CFQ) [20] was used as a self-reported measure of everyday cognitive lapses for perception, memory, and motor function, such as forgetting appointments or having word finding difficulty. The CFQ has been applied on diverse neurological and medical populations and has been shown to have appropriate psychometric properties [20]. The UCLA Loneliness Scale [21] was administered to assess subjective feelings of social isolation. The measure has established reliability and has been shown to correlate well with other measures of loneliness, and to discriminate between feelings of loneliness and depression. Finally, the EuroQoL, [22] a validated preference-based scale for which population norms are available in the US and elsewhere, was used as the global evaluation of veteran’s health-related quality of life. The EuroQoL measure combines data on activity restrictions (ADL, IADL limitations), limitations in participation (usual major activity and other social activities) and self- perceived health status (excellent, good, fair or poor) to measure one’s overall satisfaction with health and well-being.

Information was collected about the veteran’s sociodemographic and economic characteristics, including age, gender, race/ethnicity, marital status, number of children, household size, major activity/work status. These data were used to examine possible confounding variables and to control for chance differences across samples randomized to immediate and delayed entry.

#### Statistical analysis

Descriptive statistics were used to characterize the participant population and to contrast the delayed and immediate entry groups using standard *t* and χ^2^ test statistics. The main analyses, however, relied on regression-adjusted difference in means to ascertain the *independent* effect of the Guitar for Vets intervention on PTSD symptoms, depression, social functioning and quality of life. Specifically, we applied the Generalized Estimation Equation (GEE) [23, 24] regression technique to estimate intervention impacts by comparing the post-intervention experience of the entire sample (immediate + delayed entry groups) to the delay period experience (no intervention) of the delayed entry group (referred as “control” group). These GEE regressions, which adjusted for baseline levels of each outcome of interest as well as variables found to differ by chance across randomized groups, enabled us to account both for specific time-invariant effects and design clustering (repeated observations for delayed entry group veterans). Estimates of treatment-control group differences generated by these models were then tested for statistical significance to determine the intervention effectiveness of two equally motivated groups, one of which was not yet receiving active treatment.

## Results

The CONSORT flow diagram for the study is shown in Fig. 2. Of the 68 Veterans who were self- or provider-referred to the program, 25 (36.7%) were ineligible due to (i) absence of a PTSD diagnosis (*n* = 3); participation in ongoing intense psychotherapy (*n* = 4) or inpatient substance abuse program (*n* = 2); current resident of the Domiciliary (*n* = 8) and inability to attend lessons due to distance from residence to the VA (n = 8). Only 3 (4.4%) Veterans declined participation due to lack of interest. Table 1 provides descriptive information for the 40 subjects who were eligible and enrolled in the study, overall and by randomization status, as well as for the 33 (82.5%) subjects who completed the study.

**Table 1:** Sample Characteristics at Enrollment, Overall and by Randomization Group

Characteristic	At Enrollment Full Sample (*n* = 40)	At Enrollment Immediate Entry (*n* = 25)	At Enrollment Delayed Entry (*n* = 15)	Completed Follow-up Sample (*n* = 33)
Age (μ ± SD)	51.3 ± 15.0	49.8 ± 15.6	53.8 ± 14.1	50.0 ± 14.8
Male (%)	90.0	88.0	93.3	90.9
Ethnicity (%)				
Hispanic	2.5	0.0	6.7	3.0
Refused/Missing Information	10.0	12.0	6.7	9.1
Race (%)				
Caucasian/White	70.0	72.0	66.7	69.7
African American/Black	25.0	24.0	26.7	24.2
Refused/Missing Information	5.0	4.0	6.6	6.1
Marital Status (%)				
Married	45.0	40.0	53.3	48.5
Living with a partner	7.5	8.0	6.7	6.1
Separated or Divorced	30.0	24.0	40.0	30.3
Widowed	2.5	4.0	0.0	0.0
Single/Never married	15.0	24.0	0.0	15.2
Number of children (μ ± SD)	2.0 ± 1.9	1.6 ± 1.5	2.6 ± 2.4	2.0 ± 2.0
Household Size (μ ± SD)	2.2 ± 1.0	2.1 ± 0.8	2.3 ± 1.3	2.2 ± 1.0
Education (%)				
Less than high school	2.5	0.0	6.7	0.0
High school	32.5	32.0	33.3	30.3
Technical/Professional school	40.0	44.0	33.3	42.4
College degree	22.5	20.0	26.7	24.2
Refused/Missing Information	2.5	4.0	0.0	3.0
Work Status (%)				
Work full time	20.0	20.0	20.0	24.2
Work part time	10.0	12.0	6.7	9.1
Unemployed	5.0	4.0	6.7	3.0
Student	10.0	12.0	6.7	12.1
Retired, disability	45.0	40.0	53.3	39.4
Retired, non-health related	5.0	8.0	0.0	6.1
Other	5.0	4.0	6.6	6.0
War Era: OEF/OIF	27.5	20.0	32.0	30.3

The mean age of Veterans enrolled in the study was 51 years old, ranging from 22 to 76 years old. The majority was male (90%). One-quarter were African American or Black, over half were married or living with a partner, and nearly one in five had a college degree. While 30% report working full or part time, 45% were retired due to disability. Slightly over one-quarter were veterans of the OEF/OIF wars. Despite randomization, there were chance differences between the immediate (*n* = 25) and delayed entry (*n* = 15) samples with respect to ethnicity, marital status and work status, with delayed entry subjects being more likely to be married or living with a partner, Hispanic, and retired due to disability. Given the small sample size of each group, however, other nominal differences did not reach statistical significance at conventional levels. The last column of Table 1, which describes characteristics of the 33 subjects who completed the study, suggests no statistically significant differences between enrolled and completed samples with respect to socio-demographic or economic characteristics.

Table 2 shows unadjusted pre-and post-intervention differences for the overall sample as well as stratified by randomization arm (immediate and delayed entry groups). Results in Table 2 provide evidence of intervention effectiveness based on *unadjusted* outcomes. Bivariate comparisons reveal marked improvements in our primary outcome ─PTSD symptoms─ as measured by the PCLC scale (− 14.6 points or 22% reduction in symptoms for overall sample, *p* < 0.0001). These results held true for both delayed and immediate entry groups (− 11.1 and − 16.1, respectively, both significant at *p* < 0.01). Results also indicate that the intervention was effective in reducing depression symptoms (− 8.7 points or 28% reduction, *p* < 0.01 for the overall sample). Here again, the effects were large in magnitude and consistently significant across randomized groups, despite the smaller samples. The change in depression scores pre- and post-intervention was also significantly greater in magnitude and statistical significance than that observed among delayed entry veterans during the waiting period (− 8.2 points *p* = 0.0003 compared to − 4.9 points *p* = 0.02, respectively). The Guitars for Vets intervention was also effective in improving health-related quality of life as measured by the EuroQoL for the overall sample (+ 0.098 or 21% improvement relative to baseline, *p* = 0.03). These results were primarily driven by the experience of the immediate entry group who scored, on average, 29% higher post-intervention (0.134 points higher, *p* = 0.025). Similarly, self-reported cognitive difficulties were 13% lower (− 8 points, *p* = 0.006) post-intervention for the overall sample with immediate entry veterans reporting the greatest improvements (− 9.7 points, *p* = 0.009). There were no statistically significant effects on social functioning and isolation, although the direction of change was consistent with improvements.

**Table 2:** Unadjusted Enrollment, Pre- and Post- Intervention Outcomes, Overall and by Randomization Group

	Enrollment Score μ (SD)	Pre-Intervention Score μ (SD)	Change in Score^a^ (*p*-value)	Post-Intervention Score μ (SD)	Change in Score^b^ (p-value)
Post Traumatic Stress Disorder (PCL-C)					
Overall		66.7 (9.3)		52.0 (14.3)	−14.6 (< 0.0001)
Delayed Entry	69.2 (9.5)	63.7 (10.2)	−5.6 (0.06)	52.6 (15.4)	−11.1 (0.007)
Immediate Entry		67.9 (8.8)		51.8 (14.2)	−16.1 (< 0.0001)
Depression (BDI-II)					
Overall		30.3 (8.4)		21.6 (11.9)	**−8.7 (< 0.0001)**
Delayed Entry	33.3 (11.4)	29.4 (8.7)	**−4.9 (0.02)**	21.2 (9.7)	**−8.2 (0.0003)**
Immediate Entry		30.7 (8.4)		21.7 (12.9)	**−9.0 (0.004)**
Social Functioning (UCLA Loneliness Scale)					
Overall		56.1 (3.9)		54.6 (3.8)	−1.5 (0.08)
Delayed Entry	57.2 (3.0)	56.7 (3.6)	0.5 (0.66)	54.8 (2.9)	−1.9 (0.29)
Immediate Entry		55.8 (4.1)		54.5 (4.1)	−1.3 (0.17)
Quality of Life (EuroQoL)					
Overall		0.461 (0.28)		0.56 (0.22)	**0.098 (0.03)**
Delayed Entry	0.206 (0.276)	0.459 (0.30)	0.254 (0.05)	0.478 (0.26)	0.018 (0.80)
Immediate Entry		0.462 (0.28)		0.596 (0.19)	**0.134 (0.02)**
Cognitive Difficulties (CFQ)					
Overall		60.0 (17.3)		52.0 (19.9)	**−8.0 (0.006)**
Delayed Entry	62.9 (15.6)	57.8 (15.7)	**−5.1 (0.05)**	54.3 (22.3)	−4.3 (0.37)
Immediate Entry		60.7 (18.5)		51.0 (19.1)	**−9.7 (0.009)**

*P*-values forthcoming from comparison of means using two-sided paired t-tests. Differences at or below the threshold of *p* < 0.05 are marked in bold

^a^Values reflect change in score during wait period among veterans randomized to delayed entry (A_1_-X, Fig. 1)

^b^ Values reflect change in score between pre- and post-intervention periods for each group, that is, (B-A) and (B_1_-A_1_) in Fig. 1 for immediate and delayed entry, respectively

Table 3 shows *adjusted* outcomes based on coefficient estimates from the GEE models that controlled for baseline measures of the outcomes and other factors for which there were chance differences between immediate and delayed entry groups as well as repeated observations for the delayed entry group. As shown in Table 3, the average regression-adjusted PTSD severity score was reduced by 9.7 points (*p* = 0.01), or 14.3% from baseline to post Guitars for Vets. Similarly, adjusted depressive symptoms were reduced by 20.4% (− 6.3 points, *p* = 0.02). Adjusted differences for primarily to relatively large standard deviation around the point estimates obtained from the GEE models.

**Table 3:** Adjusted Intervention Effects

	Outcome				
	Post-traumatic Stress Disorder	Depression	Cognitive Failures	Social Functioning	Health-related Quality of Life
Intervention Effect	**−9.7 (*p*** **= 0.01)**	**−6.3 (*p*** **= 0.02)**	−4.4 (*p* = 0.31)	−1.9 (*p* = 0.10)	0.03 (*p* = 0.75)

Adjusted for age, gender, race/ethnicity, marital status and OEF/OIF status, variables found to differ by chance across randomized groups. All models further control for baseline values of the outcomes as well as clustering (multiple observations for individuals randomized to the delayed entry arm of the study). Statistical signficance at or below the threshold of *p* < 0.05 are marked in bold

## Discussion

Combat-related PTSD is a chronic disorder difficult to treat through pharmacological means alone; such medications can have important side effects and may not be effective in the long term. Psychotherapy and exposure based therapies remain the most empirically validated treatment options for treatment of PTSD; however, veterans are often hesitant to re-experience trauma-related emotions and struggle to express their emotions verbally. As a result, they are reluctant to engage in mental health treatment, and often find it difficult to articulate their experiences once they have engaged in such treatment. Our results, which showed significant improvements in PTSD and depressive symptoms among study participants, support the hypothesis that Guitars for Vets is an effective adjuvant therapy for emotional expression that decreases psychiatric symptoms in Veterans with moderate to severe PTSD.

With one notable exception [14], no published study has examined the effect of *active* music instruction as a strategy to improve the psychological well-being of persons with mental health issues. The Guitars for Vets intervention evaluated in our study, although unique in its conceptualization and implementation, fits squarely into the complementary and alternative medicine paradigm and has important implications for research. Adjuvant music therapy has traditionally fallen outside of empirical study and scrutiny, but is now increasingly recognized as a valuable treating modality requiring rigorous evaluation. In fact, the National Center for Complementary and Integrative Health of the U.S. National Institutes on Health (NIH) recently published its intent to fund multidisciplinary research “to develop music interventions, understand their mechanisms(s) of action, and evaluate their clinical relevance.” [25].

The study also has significant clinical implications. Veterans with PTSD tend to isolate socially; Guitars for Vets appears to provide an avenue to connect with other veterans through group-based instruction. Our findings of symptom improvement through active music participation, however, may not be solely attributed to increased social facilitation because the participants remained similar in their reported feelings of loneliness over the course of the study. Likewise, our findings are unlikely to be solely attributable to the patients becoming overall healthier because they report no significant change in health related quality of life over the study’s duration. Rather, the effect of the improvement of symptoms may relate to other factors such as increase in self-esteem by learning a new skill, introduction to a hobby in which they enjoy, or an effect of personal expression [12]. Future studies including mechanistic analyses applied to a larger and more diverse sample of Veterans with PTSD are needed to evaluate the contribution of specific intervention components or behavioral processes underlying our findings.

The population targeted for this study was vulnerable in many dimensions. Veterans with PTSD often have other injuries. Those who served in OEF or OIF report cognitive impairment even in the absence of brain injury [26]. Veterans in Domiciliary facilities also tend to be severely economically deprived and suffer from a variety of health ailments. Many have experienced homelessness and come from poor, disadvantaged communities. Our intervention was well-received despite these circumstances.

The delayed entry study design that we employed provided a robust approach to traditional contemporaneous treatment-control randomization, which was not feasible to implement as providers and investigators deemed unethical to withhold the Guitars for Vets intervention to veterans who expressed a desire to participate in the program. A similar approach has been used to overcome such ethical concerns in other settings. [27, 28] The emphasis on a multitude of validated measures of outcomes is another strength of the study. Finally, ease of recruitment and retention of participants suggest that the program is a viable option for engaging this especially vulnerable segment of the veteran population.

The study, however, is not without its limitations, chief among them the relatively small sample size afforded by this pilot program. In addition, as with any social experiment, subjects were not blinded to the intervention and outcomes were measured based on self-reported information rather than clinician-administered assessments of the underlying condition. Despite efforts to recruit women, our final sample was overwhelmingly male, limiting the generalizability of our findings to female veterans with PTSD. We were also forced to make important decisions in the design and duration of the intervention. Although the literature provides support for a multi-factorial approach and suggests that a more intensive, enduring intervention should be more effective in helping our target population, the extant research on the subject has not focused on an intervention such as Guitars for Vets and therefore does not provide clear guidance on how intensive or enduring that intervention should be. We opted to examine the effectiveness of the Guitars for Vets intervention based on the specific number of scheduled individual and group lessons currently provided by the Guitars for Vets organization. Also, given concerns about attrition, we limited the wait period for veterans randomized to the delayed-entry group to 4 weeks, two-weeks shorter than the 6-week intervention observation period for both groups. Finally, we were unable to examine the extent to which subjects continued their participation in the program once the evaluation period was over or whether the positive effects observed at 6-weeks were sustainable in the long run. Despite these limitations, our pilot study provides scientific evidence of the effectiveness of the Guitars for Vets intervention for promoting self-management of PTSD.

## Conclusion

The results of this pilot study suggest that Guitars for Vets is a safe and potentially effective intervention to improve PTSD and depressive symptoms among veterans with moderate to severe PTSD. Although a large scale study would be necessary to confirm the evidence of efficacy seen in the pilot study, and to examine its cost-effectiveness relative to usual VA PTSD care, the Guitars for Vets intervention appears to hold promise and could be promoted nationwide in VA hospitals making it policy relevant.

## Abbreviations

ADL: Activities of daily living
BDI: Beck Depressive Inventory
CAM: Complementary and Alternative Medicine
CFQ: Cognitive Failures Questionnaire
GEE: Generalized estimating equation
IADL: Instrumental activities of daily living
OIF/OEF: Operation Iraqi Freedom/Operation Enduring Freedom
PCLC: PTSD Checklist Civilian
PTSD: Post-traumatic stress disorder
VA: Veteran’s Association
